# Supplementary material for: Soil networks become more connected and take up more carbon as nature restoration progresses
Source: Nat Commun. 2017 Feb 8;8:14349. doi: 10.1038/ncomms14349 (PMC5309817; doi:10.1038/ncomms14349)
Supplement: Supplementary Information — Supplementary Figures, Supplementary Tables, Supplementary Methods, and Supplementary References [file ncomms14349-s1.pdf]

Supplementary Information

Supplementary Methods

Sequencing

Bacterial 16S rRNA gene fragments were amplified using the primer pair 530F (5'-ACTCCTACGGGAGGCAGCAG)[1] and 803R (5'-CTACCNGGGTATCTAAT-3'). The fungal ITS2 region was amplified using primers ITS4 (5'-TCC TCC GCT TAT TGA TAT GC-3') and ITS9 (5'- GAA CGC AGC RAA IIG YGA-3')[2]. Finally, bacterial and fungal amplicons were pooled at equimolar concentrations to provide a total of 2 ng DNA in each library.

Bacterial sequences were analysed using a Snakemake workflow[3] that follows the SOP for 454-sequencing data in mothur version 1.33.2[4]. The flowgrams were demultiplexed with a mismatch of 2 to the barcode, 3 mismatches to the primer, and flowgrams were trimmed to a size of 575 flows. Flowgrams were corrected using the shhh.flows command, which is the mothur implementation of the original PyroNoise algorithm[5]. Afterwards the results of the different sff files were combined for further analysis. The merged sequences were aligned and classified with SINA[6] against the SILVA 119 database[7]. Only reads that starts at position 9878 and stop at a position so that 90% of the sequence fall into this region were kept. To reduce sequence errors even more the pre.cluster command is used to merge sequences that are within 2 mismatches of each other. Chimeric sequences were identified and removed using the chimera.uchime command[8]. OTUs were formed at maximum distance of 0.03 using the dist.seqs command and average neighbour clustering. For each OTU a consensus taxonomy was determined using the classify.otu command. Representative sequences for each OTU were re-aligned to the Silva reference alignment and a neighbour joining tree was created using the clearcut program[9]. Taxonomic classification and OTU clustering data were combined into the BIOM format[10] for further downstream statistical analysis with the Phyloseq[11] package for R.

Fungal sequences were initially demultiplexed according to their multiplex identifier (MID) using the sffinfo command of Mothur v.1.22.2[4]. The raw flowgrams were filtered using the trim.flows command to a minimum flowgram length of 360 cycles before the first noisy signal (signal ranging from 0.5-0.7). All flowgrams were then truncated at 720 cycles. The internal transcribed spacer 2 (ITS2) region was extracted using Fungal ITS extractor v 2[12] and sequences shorter than 100 bp were removed. See for more details Thomson *et al.*[13].

For each microbial group, all sequences were pooled and sorted by decreasing length as recommended for Usearch clustering[14]. Operational taxonomic units (OTUs) were generated from these reads using the cluster_smallmem command at a 97 % similarity threshold. Phylogenetic assignation was done for each OTU consensus sequence using the Basic Local Alignment Search Tool (BLAST) algorithm v 2.2.23[15] against the Ribosomal Data Project (RDP) database release 10.3[16] for bacterial and archaeal sequences. For fungi, the assignation was performed using the UNITE database release 5.0[17]**.** All phylogenies were determined using a minimum e-value cut-off of 1e-5.

For bacteria and fungi, matrices containing the sequence abundances of different OTUs in each soil sample were created. To obtain comparable samples with different number of sequences, data were rarefied to least amount of reads using the 'rrarefy' comment in the vegan package in R[18]. We confirmed that the selected amount of reads was approaching the saturation end of the rarefaction curve. This rarefication procedure resulted in 2800 sequences for bacteria and 635 for fungi. The abundance of a certain OTU in a sample was used as an abundance measure for network analysis. The fungal reads were assigned into 15 ecological functional guilds (nr.31 till nr. 45) according to the Supplementary Table 5.

Supplementary References

1 Acosta-Martinez, V., Dowd, S., Sun, Y. & Allen, V. Tag-encoded pyrosequencing analysis of bacterial diversity in a single soil type as affected by management and land use. *Soil Biol Biochem* **40**, 2762-2770, doi:10.1016/j.soilbio.2008.07.022 (2008).

2 Ihrmark, K. *et al.* New primers to amplify the fungal ITS2 region - evaluation by 454-sequencing of artificial and natural communities. *FEMS Microbiol. Ecol.* **82**, 666-677, doi:10.1111/j.1574-6941.2012.01437.x (2012).

3 Koster, J. & Rahmann, S. Snakemake-a scalable bioinformatics workflow engine. *Bioinformatics* **28**, 2520-2522, doi:10.1093/bioinformatics/bts480 (2012).

4 Schloss, P. D. *et al.* Introducing mothur: Open-Source, Platform-Independent, Community-Supported Software for Describing and Comparing Microbial Communities. *Appl Environ Microbiol* **75**, 7537-7541, doi:10.1128/aem.01541-09 (2009).

5 Quince, C., Lanzen, A., Davenport, R. J. & Turnbaugh, P. J. Removing Noise From Pyrosequenced Amplicons. *BMC Bioinformatics* **12**, doi:10.1186/1471-2105-12-38 (2011).

6 Pruesse, E., Peplies, J. & Glöckner, F. O. SINA: Accurate high-throughput multiple sequence alignment of ribosomal RNA genes. *Bioinformatics* **28**, 1823-1829, doi:10.1093/bioinformatics/bts252 (2012).

7 Quast, C. *et al.* The SILVA ribosomal RNA gene database project: improved data processing and web-based tools. *Nucleic Acids Res* **41**, D590-D596, doi:10.1093/nar/gks1219 (2013).

8 Edgar, R. C., Haas, B. J., Clemente, J. C., Quince, C. & Knight, R. UCHIME improves sensitivity and speed of chimera detection. *Bioinformatics* **27**, 2194-2200, doi:10.1093/bioinformatics/btr381 (2011).

9 Sheneman, L., Evans, J. & Foster, J. A. Clearcut: a fast implementation of relaxed neighbor joining. *Bioinformatics* **22**, 2823-2824, doi:10.1093/bioinformatics/btl478 (2006).

10 McDonald, D. *et al.* An improved Greengenes taxonomy with explicit ranks for ecological and evolutionary analyses of bacteria and archaea. *Isme Journal* **6**, 610-618, doi:10.1038/ismej.2011.139 (2012).

11 McMurdie, P. J. & Holmes, S. phyloseq: An R Package for Reproducible Interactive Analysis and Graphics of Microbiome Census Data. *Plos One* **8**, doi:10.1371/journal.pone.0061217 (2013).

12 Nilsson, R. H. *et al.* An open source software package for automated extraction of ITS1 and ITS2 from fungal ITS sequences for use in high-throughput community assays and molecular ecology. *Fungal Ecology* **3**, 284-287, doi:10.1016/j.funeco.2010.05.002 (2010).

13 Thomson, B. C. *et al.* Soil conditions and land use intensification effects on soil microbial communities across a range of European field sites. *Soil Biology and Biochemistry* **88**, 403-413, doi:http://dx.doi.org/10.1016/j.soilbio.2015.06.012 (2015).

14 Edgar, R. C. Search and clustering orders of magnitude faster than BLAST. *Bioinformatics* **26**, 2460-2461, doi:10.1093/bioinformatics/btq461 (2010).

15 Altschul, S. F., Gish, W., Miller, W., Myers, E. W. & Lipman, D. J. Basic Local Alignment Search Tool. *J Mol Biol* **215**, 403-410, doi:10.1006/jmbi.1990.9999 (1990).

16 Cole, J. R. *et al.* The Ribosomal Database Project: improved alignments and new tools for rRNA analysis. *Nucleic Acids Res* **37**, D141-D145, doi:10.1093/nar/gkn879 (2009).

17 Koljalg, U. *et al.* Towards a unified paradigm for sequence-based identification of fungi. *Molec. Ecol.* **22**, 5271-5277, doi:10.1111/mec.12481 (2013).

18 R: A language and environment for statistical computing. R foundation for statistical computing, Vienna, http://www.R-project.org/. (2013).

Supplementary Figures

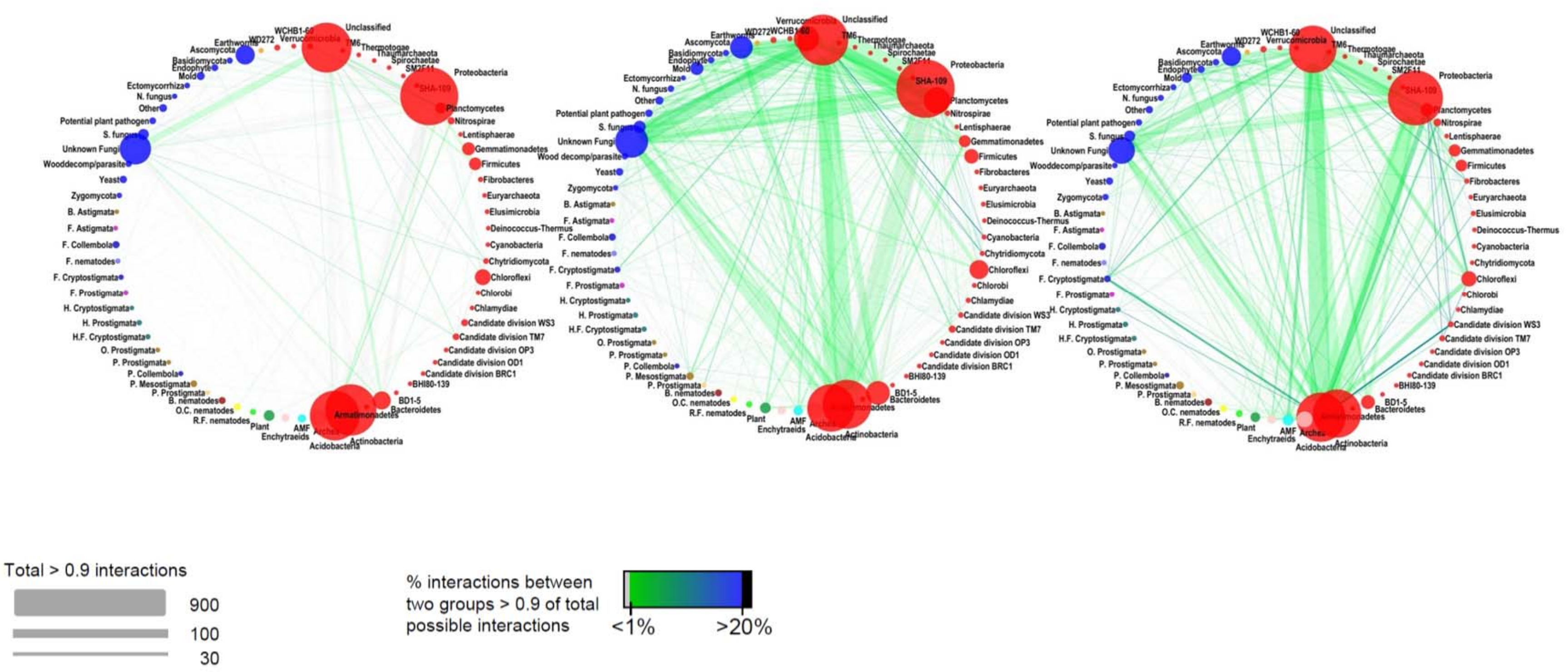

Supplementary Figure 1: Co-occurrence network visualisation sub-groups. Network visualisation of the interaction strengths between the species sub-groups in recently, mid-term and long-term abandoned agricultural fields. Spearman correlations between the co-occurrences of all individual species of different fields where calculated. The proportion of correlations > 0.9 was divided by the total number of possible interactions in order to obtain the interaction strength between two groups of species. Line width is proportional to the absolute number of correlations > 0.9. Line colour and transparency is proportional to the interactions strength as indicated in the legend. The size of the circles is proportional to the number of species/taxa in that group. Red circles are bacterial groups, blue circles are fungal groups. Abbreviations: H.=Herbivorous R.F.=Root-feeding S.=Saprotrophic F.=Fungivorous B.=Bacterivorous H.F.=Herbofungivorous N.=Nematophagous O.=Omnivorous O.C.=Omni-carnivorous P.=Predaceous.

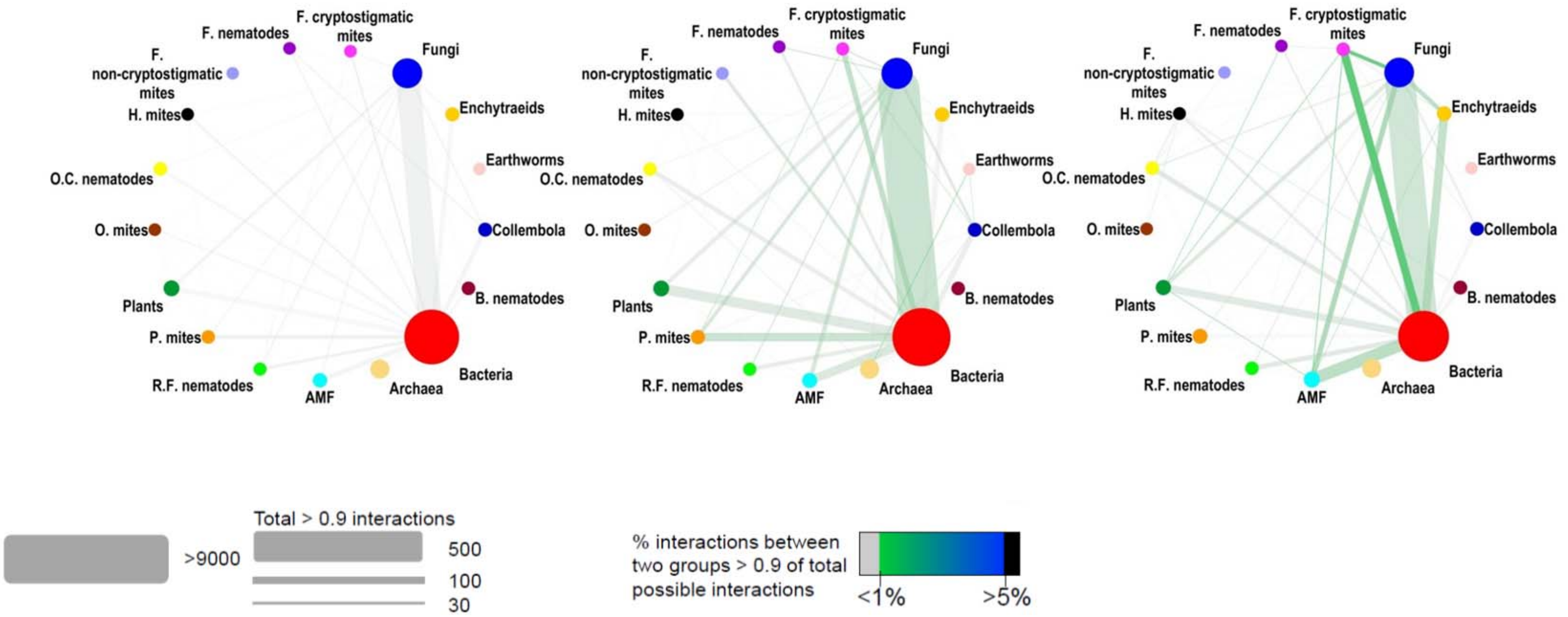

Supplementary Figure 2: Co-occurrence network visualisation main groups. Network visualisation of the interaction strengths between the main species groups in recently, mid-term and long-term abandoned agricultural fields. Spearman correlations between the co-occurrence of all individual species of different fields where calculated. The proportion of correlations > 0.9 was divided by the total number of possible interactions to obtain the interaction strength between two groups of species. Line width is proportional to the absolute number of correlations > 0.9. Line colour and transparency is proportional to the interactions strength as indicated in the legend. The size of the circles is proportional to the number of species/taxa in that group. Red circles are bacterial groups, blue circles are fungal groups. Abbreviations: H.=Herbivorous R.F.=Root-feeding S.=Saprotrophic F.=Fungivorous B.=Bacterivorous H.F.=Herbofungivorous N.=Nematophagous O.=Omnivorous O.C.=Omni-carnivorous P.=Predaceous.

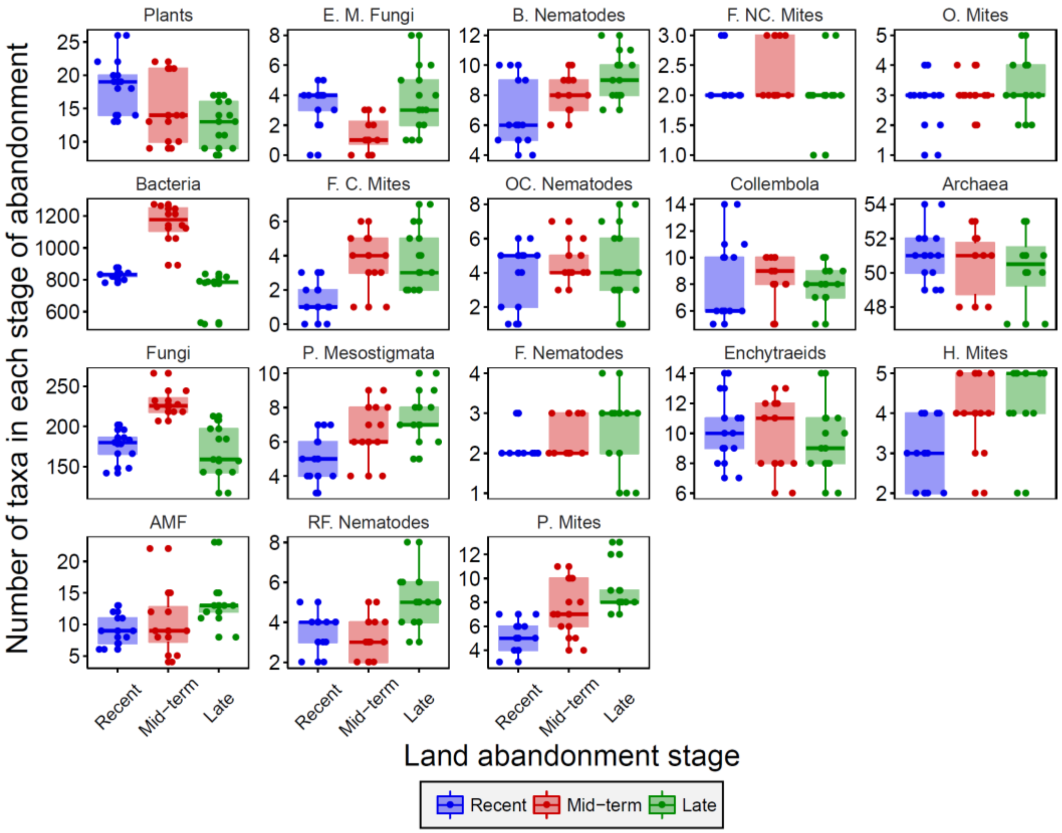

Supplementary Figure 3: Graphical representation of the Supplementary Table 5. Showing number of taxa in each stage of abandonment per sampling site. Abbreviations of field sites see site description Supplementary Table 1. Additional to Supplementary Table 5 the ectomycorrhizal fungi and predaceous mesostigmata are displayed since they show significant patterns along the successional gradient. Total amount of species within the different species groups were plotted among field sites (each bar), categories of sites (recent- (blue), mid- (red) and long-term (green) abandoned) and in years since abandonment (left to right). Line represents the median. The boxes represent the upper- and under-quartile of the data range. The whiskers represent 1.5 times the distance between the first and third quartile. Data beyond the end of the whiskers are outliers and plotted as points (as specified by Tukey). Abbreviations: E. M. fungi=Ectomycorrhizal fungi, F. C. mites=Fungivorous cryptostigmatic mites, P. mesostigmata=Predaceous mesostigmatic mites, R.F. nematodes=Root-feeding nematodes, B. nematodes=Bacterivorous nematodes, OC. nematodes=Omni-carnivorous nematodes, F. nematodes=Fungivorous nematodes, P. mites= Predaceous mites, F.NC. Mites=Fungivorous non-cryptostigmatic mites, O. mites=Omnivorous mites H. mites=Herbivorous mite.

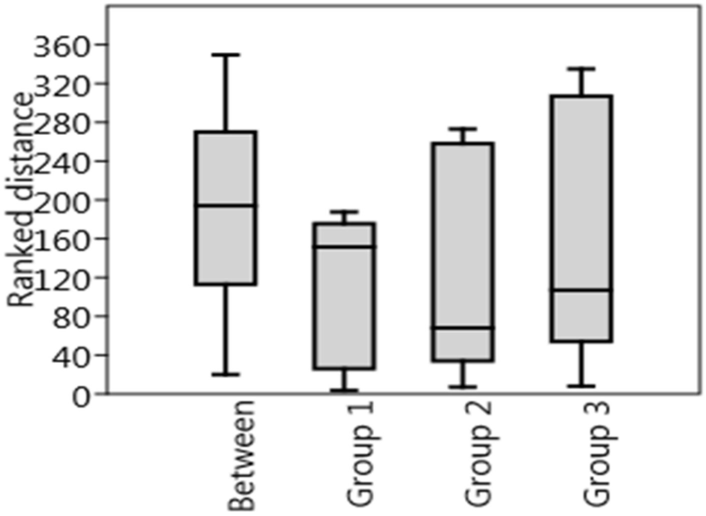

Supplementary Figure 4: Results of Analysis of similarities. ANOSIM (Euclidian, R- and p-values given) on soil properties at recent, mid- and long-term abandoned fields. The variation of the similarities between groups (recent-, mid-term-, long-term abandoned since agricultural practice, first boxplot left) is larger than the variation within groups 1, 2 and 3 representing recent-, mid-term and long-term abandonment (R=0.2333; p=0.0041). Within groups the variation of the recently abandoned fields is significantly higher than of the mid-term abandoned fields (p=0.0033), however, not significantly higher than of the long-term abandoned fields (p=0.0544).

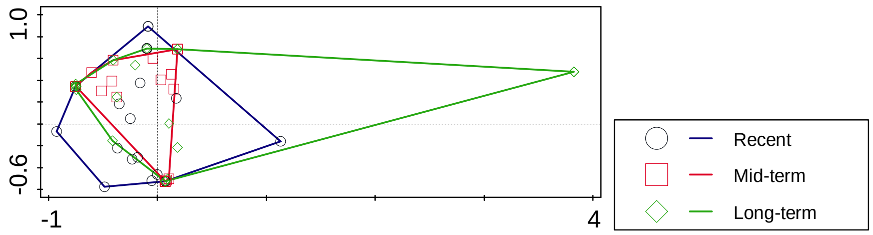

Supplementary Figure 5: NMDS of the rank abundance of plants in cores. Non-metric Multidimensional Scaling (NMDS) of the rank abundance of the three most dominated plant species (*Agrostis capillaris, Holcus lanatus, Plantago lanceolata*) in each of the experimental cores, which shows strong overlap in the plant community between the cores.

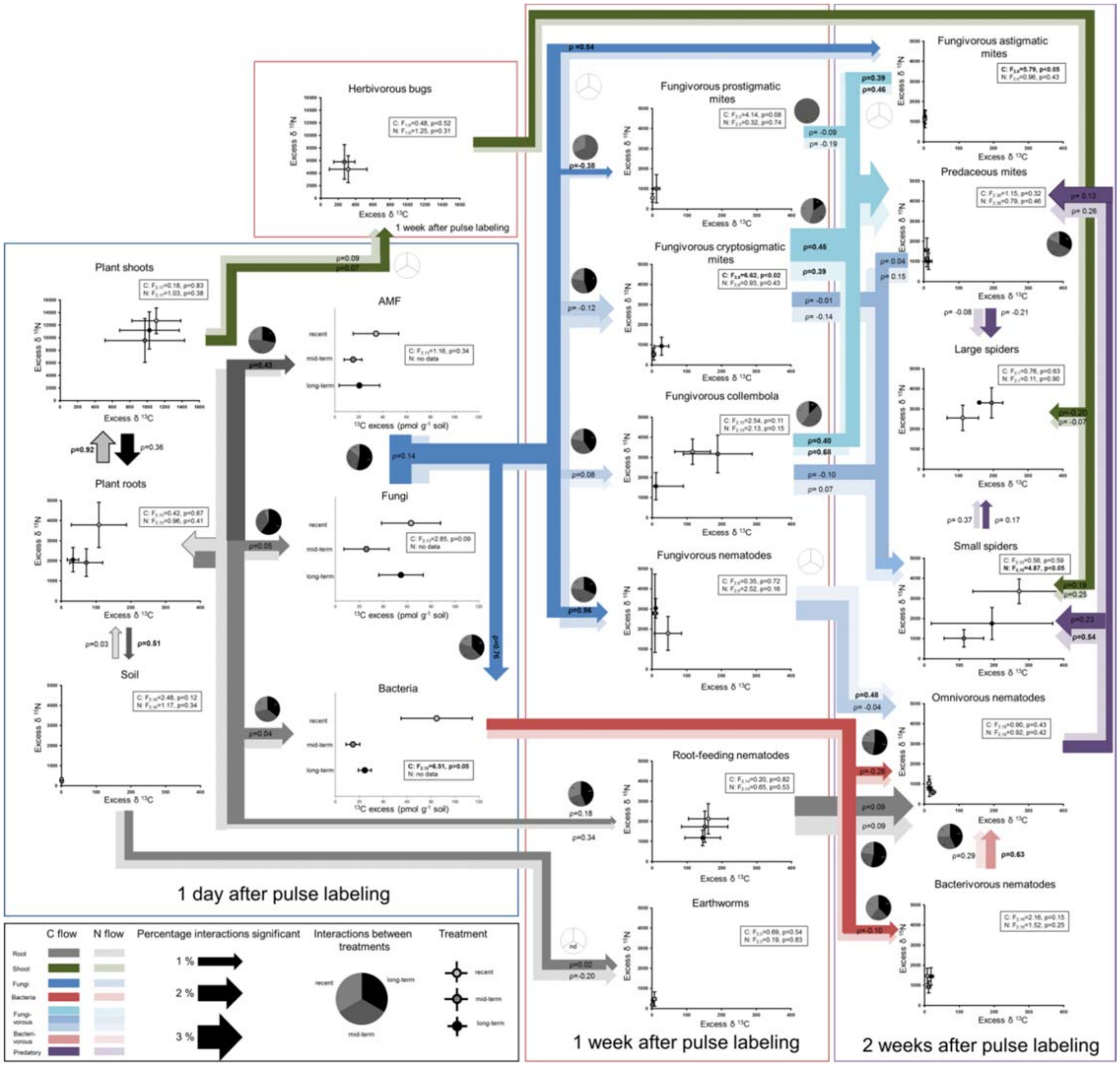

Supplementary Figure 6**:** Carbon and nitrogen flow in the soil food-web during 2 weeks. The root derived carbon is marked with grey, shoot carbon with green, bacterial-based channel with red, fungal-based channel with blue and the higher trophic level interactions with purple and pink. The plots with standard deviation represent the amount of ^{13}C excess in pmol per gram soil (bacteria, fungi, AMF), or δ^{13}C and δ^{15}N excess (all other groups) compared to natural values measured from non-labelled controls in recently (light grey), mid-term (dark grey) and long-term (black) abandoned agricultural fields. The statistical analyses of the treatment effects with field site as a factor nested in successional stage are presented in boxes next to the figure. The darker coloured arrows depict the carbon flow in the food web while the lighter coloured (dashed) arrows depict the nitrogen flow. The width of the arrows between groups reflects the percentage of correlation >0.9 between groups in all the fields as in Fig. 1. The pie charts above the arrows are also calculated from Fig. 1, and represent the proportion of significant interactions in recently (light grey), mid-term (dark grey) and long-term (black) abandoned fields. The (rho)ρ values at the arrows represent the Spearman-rank correlation values between the groups that are connected by the arrow, which is calculated from the labelling data. Significant correlations are bold and marked with darker arrow colour. By correlation analysis of pulse labelling data we wanted to analyse the potential of feeding relationships that emerged on the basis of correlations in the network analysis.

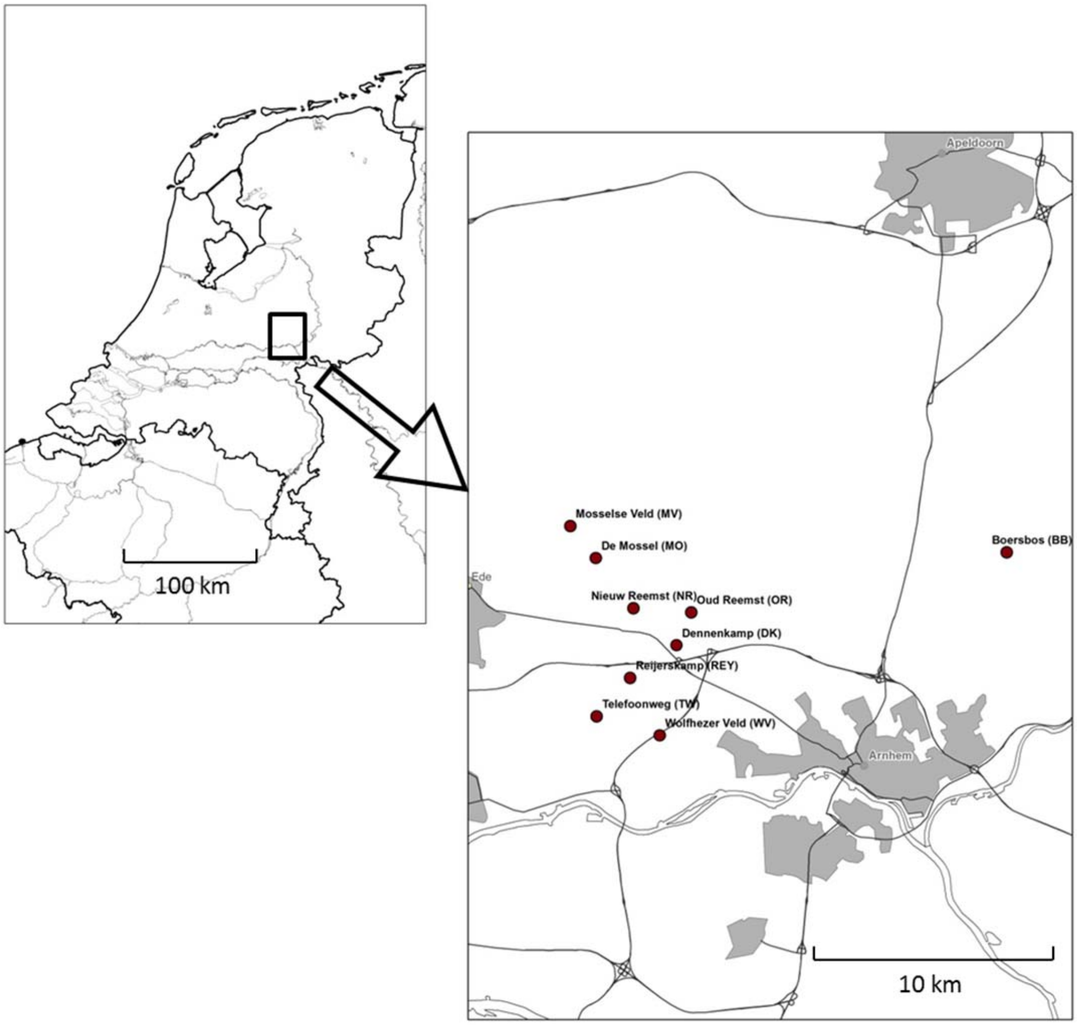

Supplementary Figure 7: Map of sampling sites in The Netherlands

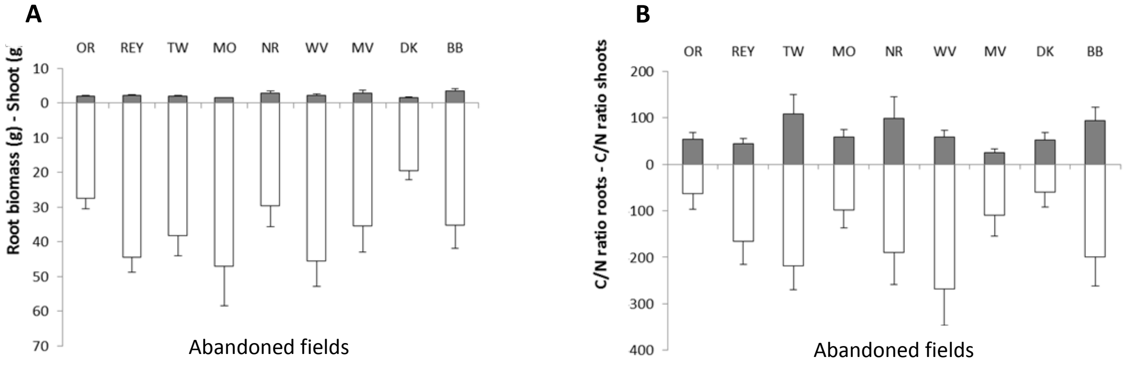

Supplementary Figure 8: Shoot + root biomass & C/N ratio of the plants. A. Shoot + root biomass in grams of the plants from the cores at the time of harvest. Abbreviations of field sites see site description Supplementary Table 1. B. C/N ratio of the plant shoots and roots from the cores at the time of harvest. Abbreviations of field sites see site description Supplementary Table 1. Error bars represent standard errors of the mean (s.e.m.).

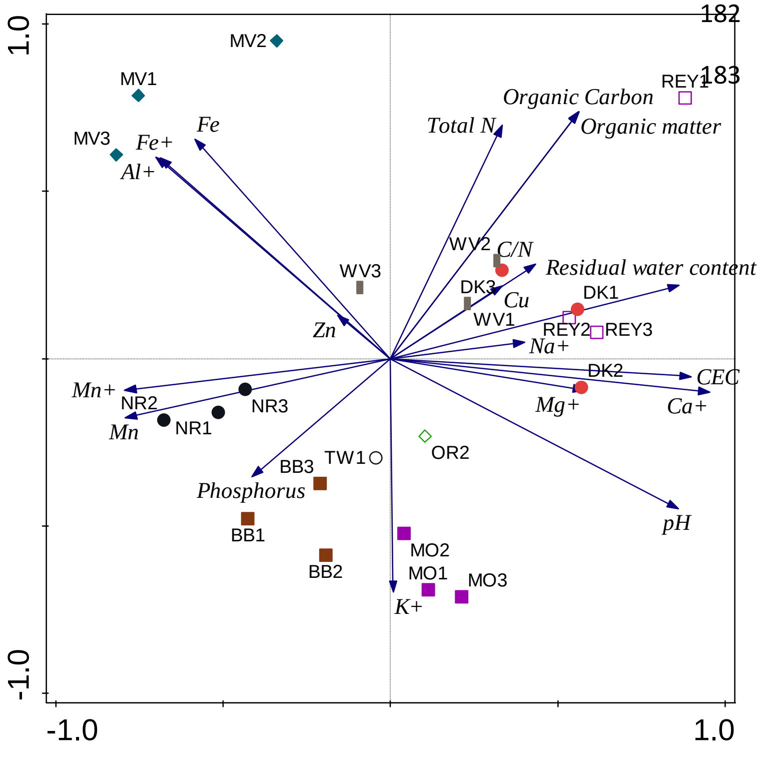

Supplementary Figure 9: PCA of site variation explained by the physiochemical data. Prinicipal Component Analysis of site variation explained by the physiochemical data collected in October 2011. Abbreviations of field sites have been provided in the site description of Supplementary Table 4. Phosphorus (P_2O_5) is measured as P-Olsen in g/kg, Cation Exchange Capacity (Cobaltihexamine) and all ions indicated with a '+' are measured as cmol/kg, Residual water content is the g/kg water remaining after drying at 105°C, metals have mg/kg as a unit, Organic Matter, Organic Carbon and Total N are measured as g/kg.

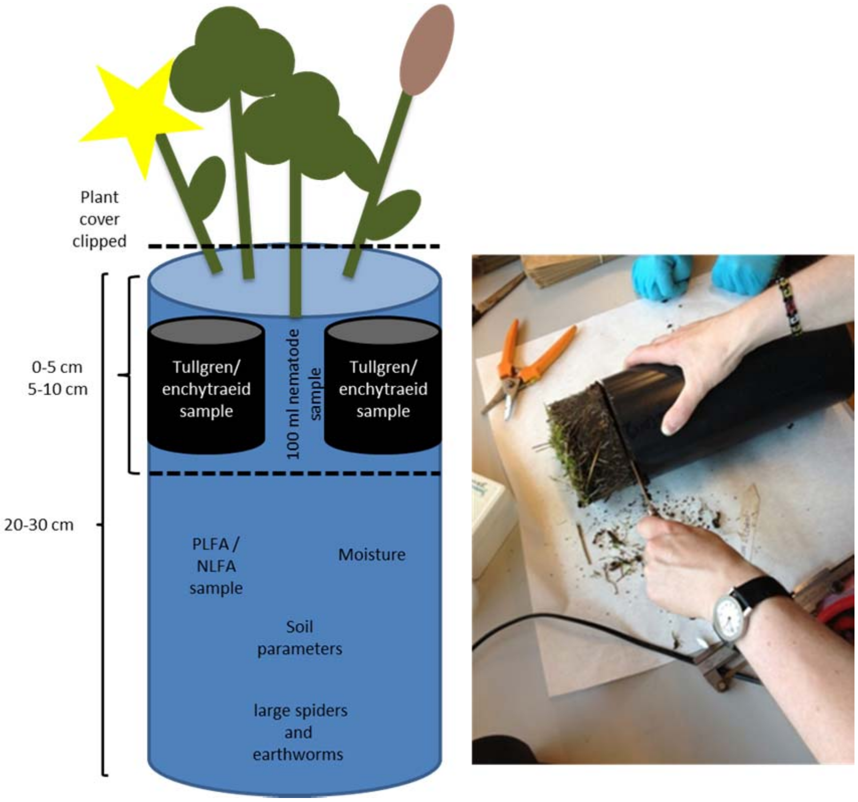

Supplementary Figure 10: Core harvesting scheme.

Supplementary Tables

Supplementary Table 1: Nested ANOVA results for Supplementary Fig. 4. Nested ANOVA results for each of the graphs from Supplementary Fig. 4. We analysed the number of species per aggregated group in three ways: the effect of site, succession, and time since abandonment. The sites OR, REY and TW (S-Table 1) were categorized as recently abandoned fields, MO, NR and WV as mid-term abandoned fields and MV, DK and BB as long-term abandoned fields. These categories mark the factor 'succession'. We also analysed the effect as a regression taking 'time since abandonment' as a continuous variable (S-Table 1). For the other factors we used a nested ANOVA approach: when testing 'site' as a factor, subplots were nested in 'site' and when testing 'succession' as a factor, sites were nested in 'succession'.

	**Site**		**Succession**		**Time since abandonment**	
	F-values	***P-values***	***F-values***	***P-values***	***F-values***	***P-values***
Bacteria	12.4	**<0.0001**	44.4	**<0.0001**	1.28	0.274
Archaea	1.11	0.412	0.348	0.712	1.06	0.316
Collembola	1.03	0.449	0.225	0.801	<0.001	0.984
Earthworms	1.96	0.112	1.86	0.185	<0.001	0.978
Enchytraeids	0.407	0.902	0.215	0.808	0.399	0.536
AMF	1.85	0.136	2.71	0.095	5.93	**0.026**
Fungi	1.3	**<0.0001**	33.8	**<0.0001**	0.156	0.698
Predaceous mites	2.2	0.078	7.51	**0.0043**	14.4	**0.001**
Fungivorous cryptostigmatic mites	8.82	**<0.0001**	14.4	**<0.001**	36.3	**<0.0001**
Omnivorous mites	0.763	0.639	0.684	0.517	1.58	0.225
Herbivorous mites	6.38	**<0.001**	9.75	**0.0014**	27.3	**<0.0001**
Fungivorous non-cryptostigmatic mites	1.4	0.262	1.4	0.272	0.072	0.792
Root-feeding nematodes	4.85	**0.003**	10.7	**<0.001**	10.5	**0.005**
Bacterivorous nematodes	2.52	**0.049**	4.84	**0.021**	9.07	**0.007**
Fungivorous nematodes	4.67	**0.003**	1.17	0.334	1.98	0.176
Omni-carnivorous nematodes	1.02	0.458	0.557	0.583	0.173	0.683
Plants	9.48	**<0.0001**	11.3	**<0.001**	31.4	**<0.0001**
Ectomycorrhiza	2.6	**0.047**	4.95	**0.02**	0.144	0.709
Predaceous mesostigmata	2.06	0.096	5.48	**0.014**	9.54	**0.006**

Supplementary Table 2: Amount of variation explained by all measured soil properties via constrained analysis. Linear (RDA) or unimodal (CCA), dependent on the length of the gradient. Then via a forward selection procedure the most explaining soil properties were selected and used to re-analyse the constrained analysis. The variation explained by each of the significant contributing soil properties is displayed below. Phosphorus (P_2O_5) is measured as P-Olsen in g/kg, Cation Exchange Capacity (Cobaltihexamine) and all ions indicated with a '+' are measured as cmol+/kg, Residual water content is the g/kg water remaining after drying at 105°C, metals have mg/kg as a unit, Organic Matter, Organic Carbon and Total N are measured as g/kg.

**Group**	**% var. expl. by all soil properties**	**Most expl. soil property 1**			**Most expl. soil property 2**			**Most expl. soil property 3**			**Most expl. soil property 4**			**Most expl. soil property 5**			**Most expl. soil property 6**		
		Env. Var.	Var. expl.	P	Env. Var.	Var. expl.	P	Env. Var.	Var. expl.	P	Env. Var.	Var. expl.	P	Env. Var.	Var. expl.	P	Env. Var.	Var. expl.	P
Bacteria	38.9% (CCA)	Fe^{+}	10.5%	0.002	Water	6.7%	0.002	Fe	6%	0.002	CEC	5.8%	0.01						
Archaea	46.1% (RDA)	Cu	12.4%	0.042															
Fungi (incl. AMF)	38.4% (CCA)	Fe^{+}	10.9%	0.002	C/N	6.2%	0.002	Fe	5.7%	0.006	P	5.3	0.036	pH	5.4%	0.022			
Protists	28.4% (CCA)	Fe^{+}	17.1%	0.002	Fe	7.9%	0.004	Cu	7.3%	0.004	P	6.1%	0.004						
Nematodes	34.6% (RDA)	Na^{+}	12.6%	0.004	Org. C	7%	0.062	Mg^{+}	7.1%	0.013	Ca^{+}	9.5%	0.036						
Enchytraeids	54.4% (CCA)	Fe^{+}	17%	0.002	Fe	7.9%	0.004	Cu	7.3%	0.004	P	6.1%	0.01	Org C	6.4%	0.004	Ca	5.0%	0.038
Mites	29.4% (RDA)	P	13%	0.016	Al^{+}	12.3%	0.018												
Collembola	48.5% (RDA)	Fe^{+}	16.6%	0.024	K^{+}	11.5%	0.05	C/N	14.4%	0.0.28									
Plants	68.4% (CCA)	pH	13.2%	0.002	Org. C	11.1%	0.002	P	7.9%	0.008	Al^{+}	6.8%	0.002	C/N	6.9%	0.006	Tot N	6.9%	0.002

Supplementary Table 3: Average label in each of the measured groups at all harvest points: 1 day, 1 week and 2 weeks after pulse labelling in atom% C. The table shows the significance levels of the three successional stages in an ANOVA with a nested design. The sites OR, REY and TW (S-Table 1) were categorized as recently abandoned fields, MO, NR and WV as mid-term abandoned fields and MV, DK and BB as long-term abandoned fields. These categories mark the factor 'succession'. Where sites are nested in succession time.

Generalized Linear Model with Nested design. Field nested in succession time

			Average	Stdev	Stats
**1 day**	Plant shoots	Recent	1.62	0.334	
		Mid	1039,00	0.482	$F_{(2,17)} = 0.18$, p=0.83
		Long	1098,00	0.363	
	Plant roots	Recent	0.119	0.086	
		Mid	0.080	0.052	$F(2,15) = 0.42$, p=0.67
		Long	0.038	0.017	
	Soil	Recent	0.000	0.000	
		Mid	0.001	0.001	$F(2,16) = 2.49$, p=0.11
		Long	0.000	0.000	
**1 week**	Earthworms	Recent	0.008	0.005	
		Mid	0.001	0.000	$F(2,5) = 0.70$, p=0.54
		Long	0.003	nd	
	Root-feeding nematodes	Recent	0.175	0.062	
		Mid	0.164	0.072	$F(2,14) = 0.20$, p=0.82
		Long	0.157	0.055	
	Fungal-feeding nematodes	Recent	0.045	0.040	
		Mid	0.003	0.004	$F(2,6) = 0.35$, p=0.72
		Long	0.009	0.003	
	Fungivorous collembola	Recent	0.125	0.056	
		Mid	0.204	0.107	$F(2,15) = 2.54$, p=0.11
		Long	0.011	0.010	
	Fungivorous cryptostigmatic mites	Recent	0.007	0.004	
		Mid	0.056	0.067	**$F(2,8) = 6.62$, p=0.02**
		Long	0.025	0.022	
	Fungivorous prostigmatic mites	Recent	0.000	0.000	
		Mid	0.012	0.010	**$F_{(4,5)} = 9.42$, p=0.02**
		Long	0.001	0.001	
	Herbivorous bugs	Recent	0.340	0.233	
		Mid	0.293	0.131	$F_{(1,6)} = 0.48$, p=0.52
		Long	nd	nd	
**2 weeks**	Bacterial feeding nematodes	Recent	0.009	0.003	
		Mid	0.013	0.007	$F_{(2,16)} = 2.16$, p=0.15
		Long	0.021	0.008	
	Fungivorous astigmatic mites	Recent	0.004	0.003	
		Mid	0.013	0.012	**$F_{(2,6)} = 5.79$, p=0.04**
		Long	0.005	0.002	
	Predaceous mites	Recent	0.008	0.007	
		Mid	0.01	0.006	$F_{(2,38)} = 0.79$, p=0.46
		Long	0.059	0.085	
	Predaceous spiders	Recent	0.126	0.012	
		Mid	0.216	0.035	$F_{(2,10)} = 0.56$, p=0.59
		Long	0.177	nd	
	Omnivorous nematodes	Recent	0.014	0.006	
		Mid	0.026	0.009	$F(2,18) = 0.90$, p=0.43
		Long	0.016	0.008	

Supplementary Table 4: Averages and standard deviations of delta ^{13}C values. Averages and standard deviations of delta ^{13}C values of unlabelled controls and labelled material of 1 day, 1 week and two weeks after pulse labelling. Numbers in bold indicate the highest numbers, the moment at which the pulse has in incorporated most ^{13}C in the tissue of the measured group of soil biota. That point was chosen to represent the label incorporated in that specific group of soil organisms.

	Non-labeled		1 day		1 week		2 weeks	
	Average	Stdev	Average	Stdev	Average	Stdev	Average	Stdev
Plant shoots	-29.54	1.28	**1002.33**	**713.03**	959.07	363.87	563.79	358.55
Plant roots	-29.78	0.57	**42.65**	**108.56**	23.42	72.20	53.36	99.01
Soil	-27.92	0.96	**-27.42**	**1.18**	-27.54	1.45	-27.08	1.38
Earthworms	-25.82	1.12	-22.94	6.60	**-22.72**	**5.31**	-22.07	3.38
Root-feeding nematodes	-27.43	5.78	80.54	96.50	**125.39**	**111.64**	125.95	172.38
Fungal-feeding nematodes	-27.94	2.35	-25.47	3.10	**-7.33**	**49.83**	-9.42	25.23
Fungivorous collembola	-23.42	1.84	57.19	62.80	**93.05**	**149.33**	81.15	106.62
Fungivorous cryptostigmatic mites	-24.63	0.27	-17.22	15.17	**-11.72**	**27.60**	-20.58	6.00
Fungivorous prostigmatic mites	-23.99	0.32	-16.20	13.21	**-18.68**	**13.20**	-19.34	8.14
Herbivorous bugs	-21.65	-	**541.14**	**963.12**	294.63	358.55	135.46	130.44
Bacterial feeding nematodes	-26.66	2.59	-16.60	27.08	-19.68	7.18	**-15.11**	**11827,00**
Fungivorous astigmatic mites	-22.45	3.76	-22.94	4.47	-23.03	3.73	**-17.38**	**13.47**
Predaceous mites	-23.84	2.52	-16.65	9.22	-14.31	18.28	**-1.66**	**86.77**
Predaceous spiders	-24.21	1.82	-24.83	1.72	167.54	262.84	**125.70**	**76.11**
Omnivorous nematodes	-24.06	9.11	-20.71	9.32	-1.656	36.39	**-6.84**	**14.56**
Enchytraeds	-27.67	-	-17.21	10.78	-39.79	-	-28.94	5.12

Supplementary Table 5: Description of field sites coordinates and time since abandonment.

Field name	Geocoordinates	Abandoned since
Oud Reemst (OR)	N 52°2’27 E 5°48’34	2005
Reijerskamp (REY)	N 52°1’0 E 5°46’21	2005
Telefoonweg (TW)	N 52°00’9 E 5°45’8	2002
De Mossel (MO)	N 52°3’40 E 5°45’8	1995
Nieuw Reemst (NR)	N 52°2’33 E 5°46’29	1990
Wolfhezer Veld (WV)	N 51°59’43 E 5°47’24	1988
Mosselse Veld (MV)	N 52°4’23 E 5°44’13	1985
Dennenkamp (DK)	N 52°1’43 E 5°48’2	1982
Boersbos (BB)	N 52°3’44 E 5°59’57	1982

Supplementary Table 6: Number of taxa within sub-group. Number of taxa within sub-group in each stage of abandonment (occurrence > 1 sample), for recent, mid-term and long-term abandoned fields, used in the Spearman correlation interaction strength network visualization (Fig. 1A)

Nr.	Names	Recent	Mid-term	Long-term	Nr.	Names	Recent	Mid-term	Long-term
1	Proteobacteria	398	403	346	31	AMF	17	22	30
2	Chloroflexi	54	69	51	32	Ascomycota	70	86	70
3	Actinobacteria	280	241	230	33	Basidiomycota	7	8	9
4	Firmicutes	38	47	34	34	Chytridiomycota	5	2	0
5	Acidobacteria	254	213	245	35	Endophytes	11	10	13
6	Verrucomicrobia	4	99	3	36	Molds	17	41	27
7	Gemmatimonadetes	40	35	36	37	Ectomycorrhiza	5	2	7
8	Nitrospirae	12	5	15	38	Nematophagous fungi	3	9	7
9	Unclassified (bacteria)	258	274	214	39	Other (fungi)	15	17	13
10	Bacteroidetes	64	91	44	40	Potential plant-pathogen	10	14	9
11	WD272	6	7	8	41	Saprotrophic fungi	30	36	31
12	Candidate_division_WS3	11	4	6	42	Unknown fungi	128	136	106
13	Planctomycetes	30	104	39	43	Wood decomposer or parasite	8	9	5
14	Candidate_division_TM7	10	12	6	44	Yeasts	13	14	13
15	Fibrobacteres	3	1	3	45	Zygomycota	4	3	8
16	SHA-109	2	2	0	46	Predaceous mesostigmata	10	14	16
17	Elusimicrobia	1	1	2	47	Herbo-fungivorous cryptostigma	3	3	3
18	WCHB1-60	1	2	1	48	Predaceous prostigmata	0	3	5
19	Thermotogae	0	0	1	49	Fungivorous cryptostigmata	3	7	10
20	Cyanobacteria	0	2	0	50	Omnivorous prostigmata	1	1	1
21	Chlamydiae	0	3	0	51	Bacterivorous astigmata	0	1	0
22	Armatimonadetes	1	2	1	52	Herbivorous prostigmata	4	4	4
23	Chlorobi	0	1	0	53	Herbivorous cryptostigmata	0	1	2
24	TM6	0	1	1	54	Fungivorous prostigmata	2	3	2
25	SM2F11	0	0	0	55	Fungivorous astigmata	0	1	0
26	Archaea	54	54	54	56	Root-feeding nematodes	6	7	10
27	Fungivorous collembola	12	12	12	57	Bacterivorous nematodes	11	11	13
28	Predaceous collembola	2	2	1	58	Fungivorous nematodes	3	4	3
29	Earthworms	2	2	2	59	Omni-carnivorous nematodes	10	8	10
30	Enchytraeids	15	18	20	60	Plants	29	29	24

Supplementary Table 7: Number of taxa within main group. Number of taxa within main group in each stage of abandonment (occurrence > 1 sample), for recent, mid-term and long-term abandoned fields, used in the Spearman correlation interaction strength network visualization (Fig. 1B).

Nr.	Names	Recent	Mid-term	Long-term
1	Bacteria	1467	1620	1286
2	Archaea	54	54	54
3	Collembola	14	14	13
4	Earthworms	2	2	2
5	Enchytraeids	15	18	20
6	AMF	17	22	30
7	Fungi	326	387	318
8	Predaceous mites	10	17	21
9	Fungivorous cryptostigmatic mites	3	7	10
10	Omnivorous mites	4	5	4
11	Herbivorous mites	4	5	6
12	Fungivorous non-cryptostigmatic mites	2	4	2
13	Root-feeding nematodes	6	7	10
14	Bacterivorous nematodes	11	11	13
15	Fungivorous nematodes	3	4	3
16	Omni-carnivorous nematodes	10	8	10
17	Plants	29	29	24
	**Subtotals**	**1977**	**2214**	**1826**
